# Structural basis of RND-type multidrug exporters

**DOI:** 10.3389/fmicb.2015.00327

**Published:** 2015-04-20

**Authors:** Akihito Yamaguchi, Ryosuke Nakashima, Keisuke Sakurai

**Affiliations:** Laboratory of Cell Membrane Structural Biology, Institute of Scientific and Industrial Research, Osaka UniversityIbaraki, Japan

**Keywords:** multidrug exporter, crystal structure, functional-rotation, ACRB, RND, multidrug resistance

## Abstract

Bacterial multidrug exporters are intrinsic membrane transporters that act as cellular self-defense mechanism. The most notable characteristics of multidrug exporters is that they export a wide range of drugs and toxic compounds. The overexpression of these exporters causes multidrug resistance. Multidrug-resistant pathogens have become a serious problem in modern chemotherapy. Over the past decade, investigations into the structure of bacterial multidrug exporters have revealed the multidrug recognition and export mechanisms. In this review, we primarily discuss RND-type multidrug exporters particularly AcrAB-TolC, major drug exporter in Gram-negative bacteria. RND-type drug exporters are tripartite complexes comprising a cell membrane transporter, an outer membrane channel and an adaptor protein. Cell membrane transporters and outer membrane channels are homo-trimers; however, there is no consensus on the number of adaptor proteins in these tripartite complexes. The three monomers of a cell membrane transporter have varying conformations (*access, binding*, and *extrusion*) during transport. Drugs are exported following an ordered conformational change in these three monomers, through a functional rotation mechanism coupled with the proton relay cycle in ion pairs, which is driven by proton translocation. Multidrug recognition is based on a multisite drug-binding mechanism, in which two voluminous multidrug-binding pockets in cell membrane exporters recognize a wide range of substrates as a result of permutations at numerous binding sites that are specific for the partial structures of substrate molecules. The voluminous multidrug-binding pocket may have numerous binding sites even for a single substrate, suggesting that substrates may move between binding sites during transport, an idea named as multisite-drug-oscillation hypothesis. This hypothesis is consistent with the apparently broad substrate specificity of cell membrane exporters and their highly efficient ejection of drugs from the cell. Substrates are transported through dual multidrug-binding pockets via the peristaltic motion of the substrate translocation channel. Although there are no clinically available inhibitors of bacterial multidrug exporters, efforts to develop inhibitors based on structural information are underway.

## Multidrug resistance and the emergence of RND efflux pumps

Multidrug resistance of pathogens and cancer cells are serious problem of modern chemotherapy. Multidrug resistance generally reflects the accumulation of many drug resistance factors, e.g., enzymes detoxifying antibiotics, mutations in drug targets and permeability barriers. Multidrug exporters are active permeability barriers and, among resistance factors, only multidrug exporters alone can cause multidrug resistance without additional factors (Blair et al., 2014). In many cases, high-level multidrug resistance in pathogens is caused by a synergetic effect of multidrug exporters and the other drug resistance factors (Bhardwaj and Mohanty, 2012).

**Figure 1:**
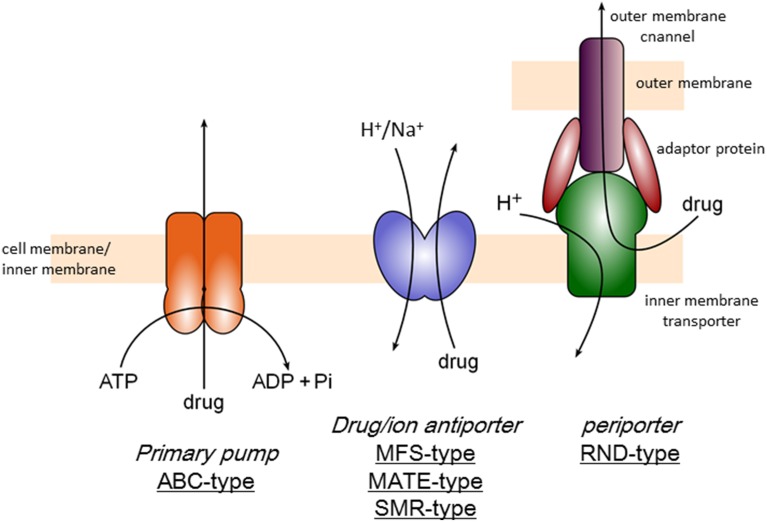
**Classification of multidrug efflux transporters**.

There are three categories of multidrug efflux transporters (Figure 1), that is, transporters driven by ATP-hydrolysis (ABC type), drug/proton or cation antiporters (MFS, MATE, and SMR-types) and tripartite transporters (RND-type), which is also drug/proton antiporter but driven by remote-conformational coupling as mentioned below. ABC (ATP-Binding Cassette)-type exporters including P-glycoprotein was first identified as a multidrug resistance factor in cancer cells (Chen et al., 1986; Gerlach et al., 1986). There are several ABC-type exporters also in bacteria and reported to contribute multidrug resistance mainly in Gram-positive organisms (Luberski et al., 2007). However, the majority of multidrug exporters in bacteria use ion motive force. MFS (Major Facilitator Superfamily)-type drug/proton antiporters (Marger and Saier, 1993) are mainly contribute to multidrug resistance of Gram-positive bacteria. SMR (Small Multidrug Resistance)-type transporters also contribute drug resistance to lipophilic drugs (Grinius and Goldberg, 1994; Paulsen et al., 1996). MATE (Multidrug And Toxic compound Extrusion)-type drug/cation antiporters including NorM contributes multidrug resistance especially in quinolone resistance in some Gram-negative pathogens (Kuroda and Tsuchiya, 2009). MATE-type transporters has also been known in mammalian cells as multidrug and toxin extrusion family (Motohashi and Inui, 2013). However, the major multidrug efflux transporters in Gram-negative bacteria are RND-type exporters (Li and Nikaido, 2015). RND-type exporters have most broad substrate specificity among bacterial multidrug exporters (Elkins and Nikaido, 2002) and the structural studies have been first advanced (Murakami et al., 2002). This review focuses the structural mechanism of RND-type multidrug export.

Gram-negative bacteria tend to exhibit higher tolerance against antibiotics than Gram-positive organisms. The reason for this antibiotic tolerance in Gram-negative bacteria was previously thought to be due to the barrier formed by their outer membranes (Nikaido, 1988). *Pseudomonas aeruginosa* exhibits the highest level of antibiotic tolerance among Gram-negative organisms. Thus, expanding the antibacterial spectrum to target *P. aeruginosa* was one of the most important early antibiotic developments. In the 1970's, porin proteins were identified as molecular sieves by which hydrophilic compounds can penetrate outer membranes (Nikaido and Vaara, 1983). The identification of carbapenem antibiotics, which are efficient against *P. aeruginosa*, represented a major milestone in antibiotic development (Slack, 1981). In the 1980's, there was controversy regarding the efficacy and pore size of the porins of *P. aeruginosa* (Hancock et al., 1979). This controversy seemed to be settled at the end of the 1980's. The pore sizes of the porins of *P. aeruginosa* were shown to be smaller than those of other Gram-negative bacteria, which are only of a sufficient size to allow the passage of monosaccharides (Yoshihara and Nakae, 1989); one of these porin proteins is specifically permeable to imipenem in molecular size exceeding the upper limit of the molecular sieve (Trias et al., 1989). However, shortly following this controversy, the drug efflux transporter MexAB was identified as a multidrug resistance factor in *P. aeruginosa* (Poole et al., 1993; Li et al., 1994a,b). Mutants that are deficient in these efflux transporters show hypersensitivity to multiple drugs, indicating that the intrinsic drug resistance of *P. aeruginosa* primarily reflects the constitutive expression of intrinsic efflux pumps (Nikaido, 1994). Small pore size of porin proteins also contributes to the drug tolerance of *P. aeruginosa* but the importance is less than efflux transporters (Li et al., 1994a). MexAB functions as a tripartite complex comprising the inner membrane transporter MexB, the outer membrane channel OprM and the adaptor protein MexA. Subsequently, in *E. coli*, AcrAB-TolC was identified as similar multidrug efflux transporter (Okusu et al., 1996) and homologs of AcrAB-TolC are found to be distributed throughout most Gram-negative bacteria (Paulsen et al., 2000). These tripartite exporters were named the RND (resistance/nodulation/division) family (Tseng et al., 1999). Several RND transporters have been identified in *P. aeruginosa* including MexAB-OprM (Li et al., 1995), MexXY-OprM (Mine et al., 1999), MexEF-OprN (Kohler et al., 1997), and MexCD-OprJ (Poole et al., 1996). In *Escherichia coli*, five RND-type drug efflux transporters have been identified (Nishino and Yamaguchi, 2001) including AcrAB-TolC and AcrAD-TolC. All of these transporters in *E. coli* couple with TolC. TolC is a multifunctional outer membrane channel (Buchanan, 2001) that not only couples with RND-type exporters but also with other types of transporters including ABC-type exporters (MacAB) (Kobayashi et al., 2001) and MFS-type exporters (EmrAB and EmrKY) (Furukawa et al., 1993; Kato et al., 2000) and the enterotoxin secretion system (Forman et al., 1995).

Clinical isolates showing multidrug resistance due to the overexpression of intrinsic multidrug exporter genes have been identified (Nikaido, 1998). RND-type multidrug exporters contribute to the multidrug resistance observed in most multidrug-resistant Gram-negative pathogens (Nikaido and Pages, 2012; Blair et al., 2014) and the inhibition of multidrug exporters restores the antibacterial activity of known antibiotics against multidrug pathogens (Pages and Amaral, 2009). Although a number of inhibitors of bacterial multidrug exporters have been developed, there has been no clinically available inhibitor until now (Bhardwaj and Mohanty, 2012). Although the physiological roles and intrinsic substrates of various types of RND-type multidrug exporters are not completely understood, these proteins have some physiological functions, beyond drug resistance 2011 (Piddock, 2006; Alvarez-Ortega et al., 2013). These proteins export intrinsic intracellular toxic metabolites, surround toxic compounds (Thanassi et al., 1997) and microbial toxins (Forman et al., 1995), and play a role in quorum sensing (Minagawa et al., 2012) and bacterial virulence (Nishino et al., 2006). Thus, intrinsic RND-type multidrug exporters likely participate in basic cellular self-defense mechanisms.

**Figure 2:**
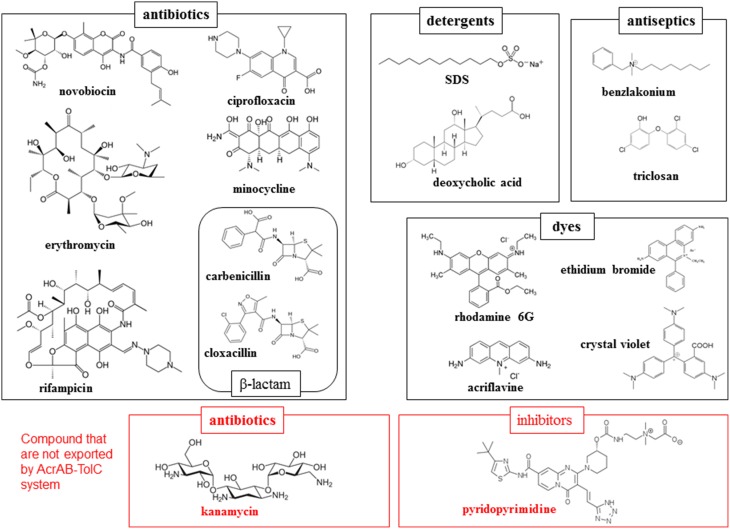
**Examples of the substrates and non-substrates of AcrAB-TolC system in *E. coli***. Black and red frames indicate the substrates and non-substrates of this exporter.

Most notably, these proteins demonstrate an extraordinarily wide substrate specificity (Elkins and Nikaido, 2002). The compounds exported through a typical RND-type exporter includes antibiotics, detergents, antiseptics and toxic dyes as well as anionic, cationic, zwitter ionic, and neutral compounds (Figure 2). These compounds also include both aromatic and aliphatic compounds. Moreover, there is no common chemical characteristic of these molecules, with the exception of amphiphilic, a characteristics of drugs and cellular toxins that assists them in moving through fluid to the target and in invading cells through the lipid bilayer of the cell membrane. However, multidrug exporters are not non-specific transporters. These proteins do not export nutrients or non-toxic metabolites such as glucose or amino acids. Additionally, these proteins transport a defined spectrum of drugs: e.g., AcrB in *E. coli* and MexB in *P. aeruginosa* do not export aminoglycoside antibiotics such as kanamycin and streptomycin, whereas AcrD in *E. coli* and MexY in *P. aeruginosa* do (Masuda et al., 2000; Elkins and Nikaido, 2002). Various inhibitors are also specific for certain multidrug exporters: e.g., pyridopyrimidine derivatives are potent inhibitors of AcrB and MexB but not MexY (Yoshida et al., 2007). Therefore, multidrug exporters recognize their substrates and are inhibited through specific mechanisms. At the start of the twenty-first century, crystal structure determinations of multidrug exporters first in RND-type (Murakami et al., 2002) followed by ABC-type (Dawson and Locher, 2006) revealed mechanisms of multidrug recognition and the active export of multidrug efflux transporters. In this review, we summarize the structural basis of multidrug recognition and active export, primarily focusing on AcrB and MexB, the most-studied RND-type multidrug exporters. We also discuss future avenues of research into the structural and mechanical aspects of multidrug exporters.

## X-ray crystallographic structure of RND-type multidrug exporter

**Figure 3:**
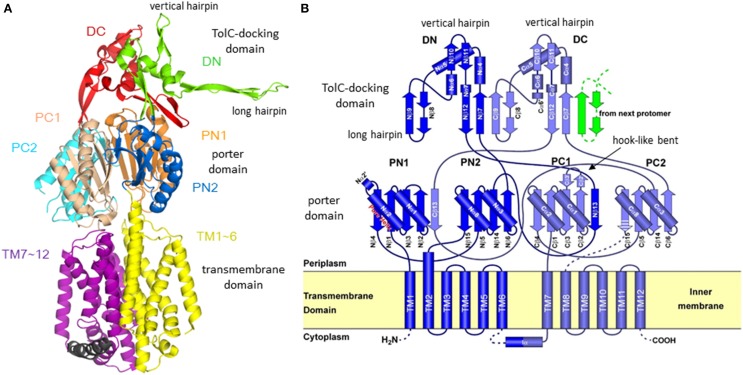
**Structure and its topology diagram of AcrB monomer. (A)** The ribbon model of AcrB monomer in three-fold-symmetric *R*32 crystal. **(B)** The topology diagram of AcrB. The hook-like bent characteristic in the C-terminal half was later identified as switching loop (Eicher et al., 2012) [or Phe617-loop (Nakashima et al., 2011)].

The first X-ray crystallographic structure of a bacterial multidrug exporter AcrB was reported by Murakami et al. (2002), showing a 3.5 Å resolution drug-free homo trimeric structure with a three-fold symmetry axis (*R*32 crystal). The monomer structure is an impressive shape like a sea horse (Figure 3A), having a long hairpin structure. The structure is divided into two parts: the transmembrane domain of approximately 50 Å in thickness and the headpiece protrudes approximately 70 Å into the periplasm. The head piece consists of two domains: the porter (or pore) domain and the TolC-docking domain. The topology diagram of AcrB monomer (Figure 3B) has a pseudo-two-fold symmetry. Each of the N- and C-terminal halves comprises six transmembrane helices, two subdomains (PN1 and PN2 or PC1 and PC2) with a β−α−β motif in the porter domain and one subdomain (DN or DC) with a short vertical hairpin protruding upward. From the DN subdomain, a long hairpin structure protrudes toward the DC domain of the next monomer.

**Figure 4:**
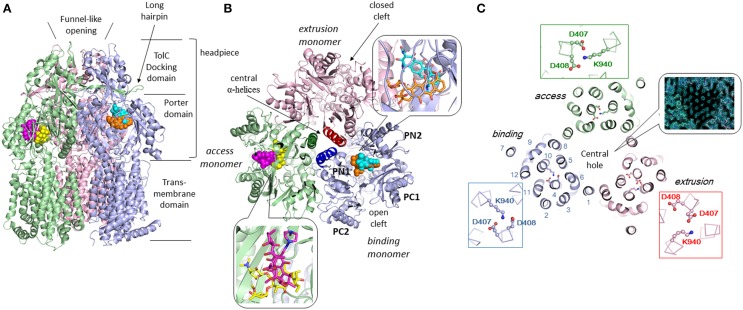
**Crystal structure of drug-binding AcrB trimers in which bound minocycline, doxorubicin, rifampicin and erythromycin overlap in the asymmetric *C*2 crystal structure (Murakami et al., 2006; Nakashima et al., 2011)**. Only one drug molecule can bind to the AcrB trimer at a time. The overall structure of the drug-free symmetric AcrB trimer in the *R*32 crystal is similar to the structure shown in this figure. The monomeric conformation of the symmetric structure is similar to the *access* monomer in the asymmetric crystal. The protein structures are shown as ribbon models. Green, blue and pink indicate *access, binding* and *extrusion* monomers, respectively. The bound drugs are depicted as space-filling models and stick models (inserts). Cyan, orange, magenta, and yellow indicate minocycline (MINO), doxorubicin (DOX), rifampicin (RIF), and erythromycin (EM), respectively. **(A)** Side view. **(B)** Horizontal cut view of the porter domain with magnified drug binding sites (insert). PN1, PN2, PC1, and PC2 are subdomains of the porter domain showing pseudo-symmetric β−α−βmotifs. The three central α-helices (one for each monomer) are highlighted in dark colors. **(C)** Horizontal cut view of the transmembrane region of the asymmetric AcrB trimer. Asp407, 408, and Lys940, which form ion pairs in the transmembrane core region, are depicted as ball and stick models. The insert shows the electron density observed in the transmembrane hole at the center of the MexB transmembrane trimers, indicating the hole is filled with a phospholipid bilayer (Nakashima et al., 2013).

**Figure 5:**
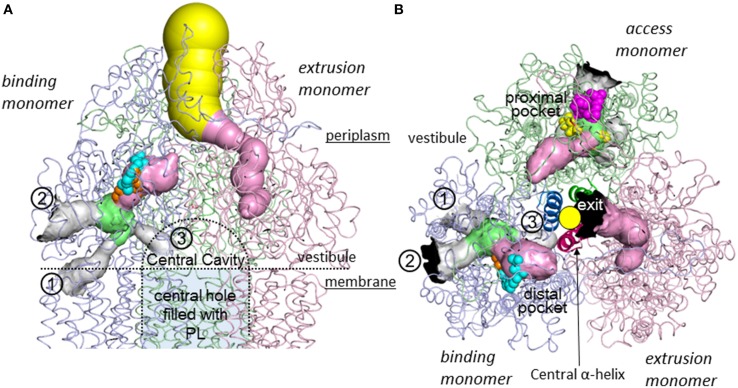
**Intramolecular water-accessible channels in the AcrB trimer (Nakashima et al., 2011)**. The channels are shown as colored solid surfaces, as calculated using the CAVER program (Medek et al., 2007). The proximal pocket, distal pocket, entrances and funnel-like exit are depicted in green, pink, gray, and yellow, respectively. The channel apertures at the entrance and exit are depicted in black. (1) Inner membrane entrance (Murakami et al., 2006), (2) periplasmic entrance (Seeger et al., 2006), (3) central cavity entrance (Nakashima et al., 2011). **(A)** Side view. The channels in the *access* monomer behind the figure have been omitted. The central cavity and central hole are depicted as dotted lines. **(B)** Horizontal cut view of the porter domain. The yellow circle indicates the closed pore-like structure comprising three central α-helices (depicted as a ribbon model with dense color), which was postulated to be a part of the putative substrate translocation channel during the early stages, however, it was not the case. The central α-helix of extrusion monomer is inclined 15° toward binding monomer more than the other two α-helices and, as a result, blocks the exit from the drug binding site. Bound minocycline (cyan), doxorubicin (orange), rifampicin (magenta), and erythromycin (yellow) overlap in the space-filling model.

**Figure 6:**
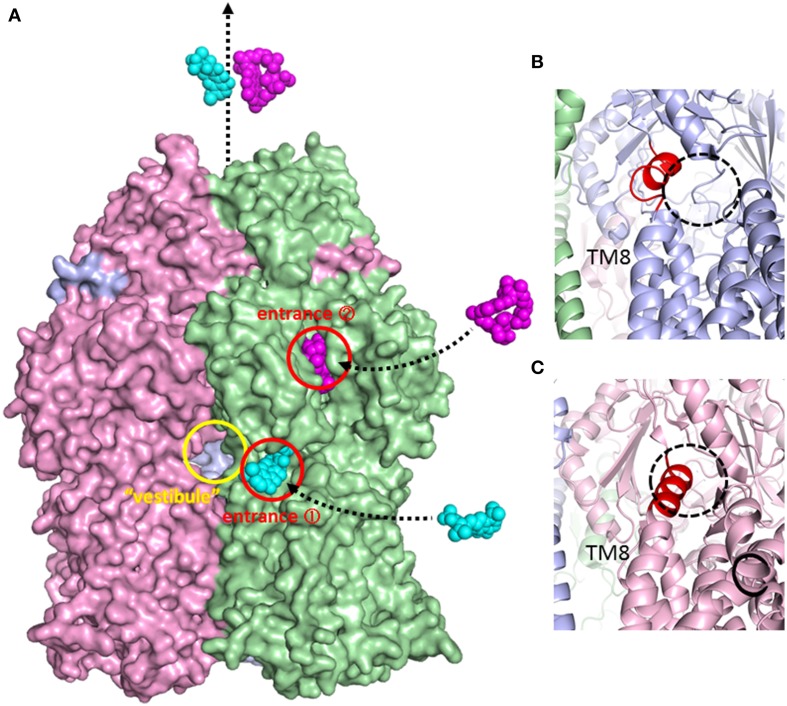
**Surface model of the AcrB trimer and magnified view of the inner membrane entrance. (A)** Side view of the AcrB trimer surface model. *Access, binding*, and *extrusion* monomers are depicted in green, blue and pink, respectively. The entrances are shown as circles. Minocycline (cyan) and rifampicin (magenta) are illustrated in space-filing models in the putative drug export route. The “vestibule” indicates window of the central cavity. **(B)** Open inner membrane entrance (entrance 1) of the *binding* monomer. The untied random coil upon N-terminal of TM8 is depicted in red. **(C)** The closed inner membrane entrance (entrance 1) of the *extrusion* monomer. The extended α-helix at the N-terminus of TM8 is depicted in red.

Three monomers of AcrB are tightly interacted to form a jelly-fish or mushroom-like structure (Figure 4A). Long hairpins are deeply inserted into the next monomers to form the shape that is likened to the figure called “it takes three to tango” (Lomovslaya et al., 2002). Three PN1 subdomains form a core for the headpiece and the three central α-helices form a pore-like structure at the center of the trimer (Figure 4B). The funnel-like 30 Å opening at the top of the TolC-docking domain is the same size as the bottom of the TolC channel (Koronakis et al., 2000). In the transmembrane domain, there is a central hole of 30 Å diameter surrounded by three 12-α-helix bundles (Figure 4C). The central hole is not a water-filled channel: it is filled with a phospholipid bilayer (Nakashima et al., 2013). There is a central cavity on the putative surface of the phospholipid bilayer in the central hole and below the closed pore-like structure (Figure 5A). The central cavity comprises three windows, named vestibules, to the surface of the inner membrane (Figures 5, 6). Initially, this central cavity was identified as a substrate binding site, at which the substrates are taken up through vestibules from the outer leaflet of the phospholipid bilayer membrane, bound to the central cavity, transferred through the central pore when it is open, and ultimately extruded from the funnel-like exit at the top of the AcrB trimer into the TolC channel (Murakami et al., 2002). Although the substrate-binding structures of the three-fold symmetry crystals in the central cavity were reported in 2003 (Yu et al., 2003), subsequent studies have not been able to identify significant electron densities of bound drugs in the central cavity [Pos et al. (2004); Murakami et al. (unpublished observation)] of the symmetric crystal.

## Functional-rotation mechanism of drug efflux

The physiologically-relevant drug-binding structures of AcrB was determined using C2 crystals, which have no three-fold symmetry (Murakami et al., 2006) (see Figure 4, which is the overlay structure of the four drug-bound AcrB structures later determined). Initially, the bound drug was identified using a bromine derivative of minocycline. Unlike symmetric crystals that show three or more bound substrates (Yu et al., 2003, 2005; Drew et al., 2008; Hung et al., 2013), only one drug molecule was bound to the AcrB trimer. The three monomers have different conformations from each other, representing three major steps of drug export, that is, *access, binding*, and *extrusion*. The minocycline or doxorubicin molecule bound not to the central cavity (Figure 4) but in the phenylalanine-rich pocket at the center of the porter domain between PC1 and PN2 of *binding* monomer (Figure 4B). An intramolecular water-accessible channel continued from entrances to the minocycline binding pocket (Figure 5). One entrance (entrance 1 in Figure 5) opens to the outer layer of the inner membrane. This inner-membrane entrance is in the vicinity of the “vestibule” that was previously identified as a drug entrance to the central cavity, but the inner-membrane entrance is distinct from this vestibule (Figure 6A). The inner-membrane entrance shows opening and closing movements during drug transport (Figures 6B,C): however, the previously identified “vestibule” is constitutively open. The channel is interrupted at the distal end of the drug-binding pocket by steric hindrance via a central α-helix of the extrusion monomer (Figure 5B), which is inclined to block the exit from the drug-binding pocket of the *binding* monomer. Thus, the *binding* monomer is in an inside-open form. Notably, the closed-exit conformation of the *binding* monomer reflects the cooperation of the next monomer.

The conformation of the monomer next to the *binding* monomer showed an outside open form. That is, the inner membrane entrance (entrance 1) is closed because of the elongation of N-terminus of TM8 (Figure 6C). The periplasmic entrance (entrance 2) is also closed because the PC2 subdomain swings toward the PC1 subdomain and the outside cleft is closed (Figure 4B). In contrast, the exit is open because the central α-helix is inclined at 15° away from the exit toward the *binding* monomer and because the PN1 subdomain swings away from the PN2 subdomain (Figures 3B, 4B). Additionally, the binding pocket shrinks because PC1 swings toward PN2. Thus, the bound substrate is squeezed from the binding pocket into the central funnel. This outside-open monomer was identified as the *extrusion* monomer.

The third monomer also has an inside open conformation similar to the *binding* monomer with the exception that the binding pocket is not expanded. Thus, the third monomer is referred to as the *access* monomer. Drugs are transported through concerted sequential conformational changes: *access, binding* and *extrusion* (Figures 3B, 4B). The conformational changes of the monomers are inter-dependent, and no two monomers have the same conformation at the same time. This process is referred to as a functional-rotation mechanism (Murakami et al., 2006) (**Figure 8**).

Symmetric forms of AcrB trimers likely reflect “resting forms”; the conformations of the three symmetric monomers are similar to the *access* monomer of the asymmetric trimers. Regarding the drug-binding symmetric crystal, the bound drugs may not reflect the physiological function. Most likely, the resting form without a substrate is symmetric, and drug binding triggers the conformation change to the asymmetric, functional form. The crystallized asymmetric structure of MexB without drugs contained bound detergent in the drug-binding pocket (Sennhauser et al., 2009). The drug-free asymmetric structure of AcrB may contain a detergent or an intrinsic substrate that has not yet been identified. Most of substrates including drugs, that bind to the AcrB crystal, are difficult to identify because these substrates may be disordered.

Seeger et al. (2006) independently reported the asymmetric structure of drug-free AcrB and Sennhauser et al. (2007) reported the structure of DARPin-bound AcrB. Asymmetric structures have also been reported for MexB (Sennhauser et al., 2009). Functional-rotation mechanism is also supported by the experiments using covalently-linked AcrB trimer (Takatsuka and Nikaido, 2009) and the experiments using engineered disulfide bonds (Seeger et al., 2008). The functional-rotation mechanism including two or more binding monomers were also proposed (Pos, 2009; Ruggerone et al., 2013) mainly due to explain bi-site activation. However, bi-site activation can be explained by peristaltic mechanism via two drug binding pockets mentioned in the next section. Symmetric drug-binding structures, which have been reported so far (Yu et al., 2003, 2005; Drew et al., 2008; Hung et al., 2013), are completely different from the *binding* monomer of functional-rotation cycle, and no pseudo-two-fold symmetric crystal structure comprising two *binding* monomers or two *access* monomers has been identified. If two of the three monomers have the same conformation at any given moment, then the structural inter-dependence between these monomers will be loose, and one monomer can independently transport drugs. Takatsuka and Nikaido (2009) verified the strict functional-rotation mechanism using covalently linked AcrB trimers that function in intact cells. When one of the three monomer units in the covalently-linked trimers was inactivated through mutations in the proton relay network in the transmembrane region or through disulfide cross-linking of the external cleft in the periplasmic domain, the entire trimeric complex was inactivated. These results clearly indicate that each monomer does not work independently. We believe that each monomer works strictly in concert with the other monomers and all monomers have different conformations in each other in any moment at the active state until pseudo-two-fold symmetric structure of AcrB will be identified.

## Multi-pocket multisite drug binding with multiple-entrances and the peristaltic mechanism of drug efflux

**Figure 7:**
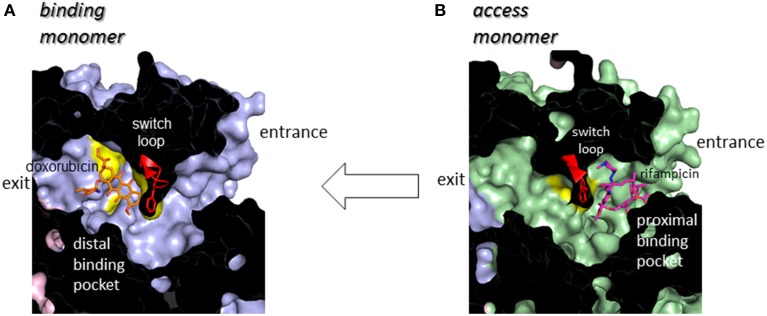
**Cut view of the transmembrane channels of the *binding* monomer (A) (blue) with bound doxorubicin (orange) and that of the *access* monomer (B) (green) with bound rifampicin (magenta)**. The switch loop containing Phe617 at the tip is depicted using a red ribbon model.

Following minocycline and doxorubicin-binding structures, the drug binding structures of AcrB bound to the high molecular mass drugs (HMMD) rifampicin and erythromycin were reported (Nakashima et al., 2011). Similar to the low molecular mass drugs (LMMD) minocycline and doxorubicin, one molecule of rifampicin or erythromycin bound to one AcrB trimer. However, rifampicin and erythromycin binding monomer was not a *binding* monomer but an *access* monomer (Figures 4A,B). The rifampicin- and erythromycin-binding pocket is located between PC1 and PC2 in the substrate translocation channel between the entrance(s) and the LMMD binding pocket (Figure 5). Thus, the HMMD-binding pocket is referred to as a proximal pocket, and the LMMD-binding pocket is referred to as a distal pocket. Shortly after the report of proximal drug binding (Nakashima et al., 2011; Eicher et al., 2012) independently reported the presence of the proximal pocket in the *access* monomer in which the doxorubicin dimer is bound. The proximal pocket in the *access* monomer is voluminous permitting typical multisite-binding of the HMMDs rifampicin and erythromycin (Figure 4B insert). However, in *access* monomer, the distal pocket is smaller than the proximal pocket. In contrast, in the *binding* monomer, the distal pocket expands and the proximal pocket shrinks. Both pockets are separated by a switch loop (Figure 7). The path under the switch loop is too narrow for the HMMDs to move into the distal pocket. The switch loop swings during the conformational change from the *access* stage to the *binding* stage. HMMDs could be transferred from the proximal pocket to the distal pocket through the swinging of the switch loop and proximal pocket shrinking, followed by distal pocket expansion during the transition from the *access* to the *binding* stages (Nakashima et al., 2011). The importance of switch loop flexibility in export is supported by the fact that when site-directed mutagenesis fixes the loop through the introduction of double proline residues into the loop (Nakashima et al., 2011) or a G616N mutation (Cha et al., 2014), the resultant mutants have completely lost or significantly decreased the drug export activity. The crystal structure revealed that a double proline mutation fixed the loop conformation at a state between the *access* and *binding* stages (Nakashima et al., 2011).

The roles of both pockets have been demonstrated through using site-directed mutagenesis. The resistance of AcrB-expressing *E. coli* cells to erythromycin is not only completely lost after site-directed mutagenesis in the proximal pocket but also reduced through mutations in the distal pocket. Doxorubicin export activity is lost through mutations in the distal pocket but remains unaffected by proximal mutations. Doxorubicin export is competitively blocked not only by the distal-binding drug minocycline but also by the proximal-binding drugs erythromycin and rifampicin (Nakashima et al., 2011). These observations indicated that both HMMDs and LMMDs are transported through both proximal and distal pockets during export.

**Figure 8:**
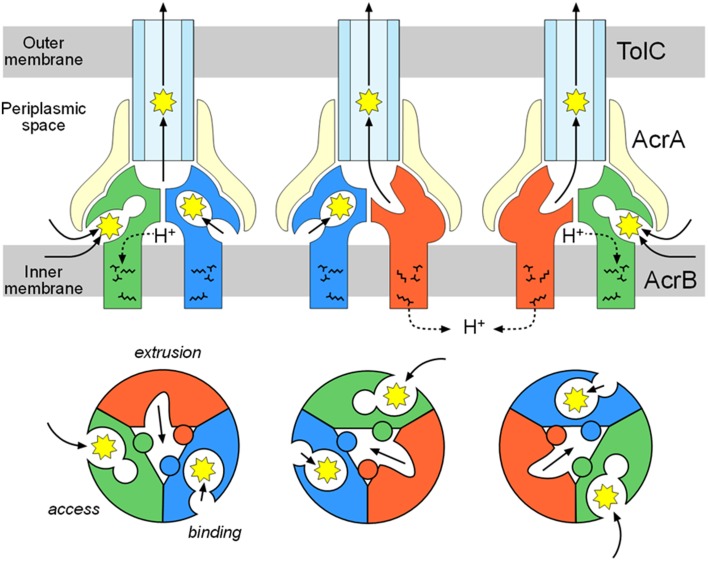
**Functional-rotation mechanism of drug export mediated through the AcrAB-TolC tripartite complex**. The upper and lower panels show side and horizontal views, respectively. The green, blue and red colors indicate the *access, binding* and *extrusion* stages of AcrB, respectively. The yellow and pale blue colors indicate AcrA and TolC, respectively. The jagged circles indicate substrates.

The double drug-binding AcrB trimer structure (in which rifampicin binds in the proximal pocket of the *access* monomer and minocycline binds in the distal pocket of the *binding* monomer) was determined. No drugs were detected in the distal pocket of the *access* monomer or the proximal pocket of the *binding* monomer, indicating that the proximal pocket and distal pocket are only activated during the *access* stage and the *binding* stage, respectively (Nakashima et al., 2011). Now the story of drug export is as follows: drugs initially enter the proximal pocket at the *access* stage. At this stage, HMMDs are bound and recognized in the expanded proximal pocket, but LMMDs are hardly or only weakly bound. Subsequently, the drugs are transferred to the distal pocket through the swinging of a switch loop and the relative motion of the subdomains, PC2, PC1, and PN2, resulting in a reduction in the volume of the proximal pocket and the expansion of the distal pocket during the transition from the *access* stage to the *binding* stage. LMMDs are bound and recognized in the distal pocket: HMMDs are not tightly bound but are instead occluded in the distal pocket because the path under the switch loop is too narrow to permit the return of HMMDs to the proximal pocket. Ultimately, the drugs are squeezed out through the TolC channel via a funnel-like opening as a result of conformational changes at the *extrusion* stage. In other words, drugs are moved through the intramolecular channels by a peristaltic motion of the two tandem drug-binding pockets (Figure 8). Multi-pockets having different substrate-binding specificity contribute to expand substrate spectrum.

An AcrB trimer with one drug bound to the *access* monomer and a second drug bound to the *binding* monomer may form the structural basis for the reported allosteric bi-site activation of the AcrAB-TolC pump (Seeger et al., 2006; Pos, 2009). Notably, the dual drug-binding structure does not indicate the presence of two *binding* monomers in one trimer. The two drug binding structures differ: one is an *access* monomer and the other is a *binding* monomer. There is no structural evidence for the presence of two or three *binding* monomers in one trimer.

Sennhauser et al. (2007) reported two possible substrate entrances: the inner-membrane entrance (entrance 1 in Figure 5) and the periplasmic entrance (entrance 2 in Figure 5). The inner-membrane entrance is the entrance described by Murakami et al. (2006). The periplasmic entrance is open to the periplasm at the bottom of the cleft between PC1 and PC2. Site-directed mutagenesis revealed that both entrances perform drug export (Husain et al., 2011; Nakashima et al., 2011). The third possible entrance (entrance 3 in Figure 5) from the central cavity is also identified (Nakashima et al., 2011): however, there is no evidence that the entrance 3 has any functions. The channels from all three putative drug entrances are merged at the proximal pocket. Molecular simulation studies showed that the inner-membrane entrance and the periplasmic entrance play a role in the export of hydrophobic compounds and hydrophilic compounds, respectively (Yao et al., 2013). Thus, it is reasonable to assume that the outer-layer entrance takes up hydrophobic drugs with relatively low-molecular-mass from the outer layer of the inner membrane (thereby acting as a membrane “vacuum cleaner” mechanism) and that the periplasmic entrance takes up hydrophilic drugs with relatively large molecular mass from the periplasm (acting as a periport). Multi-entrances contribute to expand the physico-chemical characteristics of the substrates.

## Structural basis of multidrug recognition

The induced-fit mechanism is one potential mechanism for the enzymatic recognition of multiple substrates with different chemical structures (Vogt et al., 2014). In this mechanism, the size of the substrate binding site and/or the arrangement of amino acid side chains in the binding site changes with the chemical structure of the substrates. In AcrB, the doxorubicin and minocycline binding structures of the distal pocket of the *binding* monomer, and the rifampicin and erythromycin binding structures of the proximal pocket of the *access* monomer, are not significantly different from each other with the exception of some minor changes in the orientation of the side chains directly interacting with the bound drugs. Thus, although the protein structures of AcrB, including the drug translocation channel, changes considerably during the functional-rotation cycle, the induced-fit mechanism is not the primary mechanism of multidrug recognition.

Multisite drug binding is another potential mechanism of multidrug recognition as has been described for the multidrug-binding transcription regulator QacR (Schumacher et al., 2001). In this mechanism, as mentioned above, the substrate-binding pocket is voluminous, permitting the presence of numerous binding sites for various substrates. The pocket possesses numerous binding sites that correspond to the partial structures of various compounds. The substrates are recognized through permutations of these binding sites. Although minocycline and doxorubicin have a common tetracyclic structure, the binding site of doxorubicin only partially overlaps with that of minocycline in the distal pocket (Figure 4B insert). Doxorubicin and minocycline interact with almost different sets of amino acid side chains. Rifampicin and erythromycin show the similar multisite binding in the proximal pocket (Figure 4B insert).

Recent molecular dynamics simulations have revealed that a number of structurally-distinct drugs bind to a number of sites that may slightly or substantially differ in the voluminous binding pocket of AcrB (Takatsuka et al., 2010; Vargiu and Nikaido, 2012). Additionally, the presence of two voluminous multisite drug-binding pockets, the proximal and distal pockets, with different substrate specificities greatly contributes to expanding the specificity. Multiple entrances also expand the drug specificity by facilitating drug uptake from two physically different spaces (the outer leaflet of the inner membrane and the periplasm).

**Figure 9:**
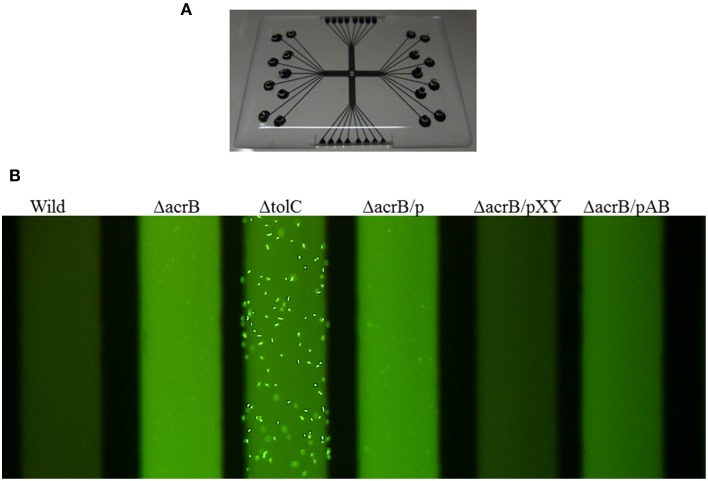
**Fluorescence assay of drug ejection from the cell by multidrug exporters (Matsumoto et al., 2011). (A)** Microfluidics device used in this experiment. *E. coli* cells and the pre-fluorescent dye FDG were mixed and injected to the wells of the device. After incubation, microfluidics tubes were observed by a fluorescence microscope. Upon entering the cytoplasm, FDG is hydrolyzed by intrinsic β-galactosidase and fluorescein is produced. When fluorescein accumulates in the cytoplasm, the cells fluoresce. When fluorescein is exported from the cytoplasm, the medium fluoresces. **(B)** Fluorescence of the microfluidics tubes. Wild: wild-type *E. coli* MG1655 cells, ΔacrB: *acr*B-deficient cells, ΔtolC: *tol*C-deficient cells. ΔacrB/p, ΔacrB/pXY, ΔacrB/pAB indicate *acr*B-deficient cells transformed with vector (pMMB67HE) and the plasmids recombined with *P. aeruginosa* efflux pump genes *mex*XY-*opr*M and *mex*AB-*opr*M, respectively (Matsumoto et al., 2011).

Identifying bound drugs in the asymmetric AcrB structure is difficult for most substrates and inhibitors. A drug may not tightly bind to specific sites. However, multidrug exporters exhibit strikingly high efficiency at rejecting substrates. When multidrug exporters are sufficiently expressed, most drug molecules are rejected prior to entering cytoplasm. Experiments using fluorescent dye (Figure 9) have shown the drug rejection efficiency of multidrug exporters (Matsumoto et al., 2011). FDG is a pre-fluorescent compound that has no fluorescence itself but demonstrates fluorescein emission when hydrolyzed by intrinsic β-galactosidase in *E. coli*. However, when FDG was added to wild-type *E. coli* cells, almost no fluorescence was observed. In *acr*B-deficient *E. coli* cells, the entire medium was fluorescent, likely reflecting the export of fluorescein from the cell through other RND-type drug exporters that do not completely reject FDG. When *tol*C was deleted, all RND-type exporters became inactive, and the cell body showed strong fluorescence because of fluorescein accumulation in the cell. However, how is this efficient rejection consistent with the apparently weak binding affinity of drugs to multidrug exporters, as predicted in crystallographic studies?

**Figure 10:**
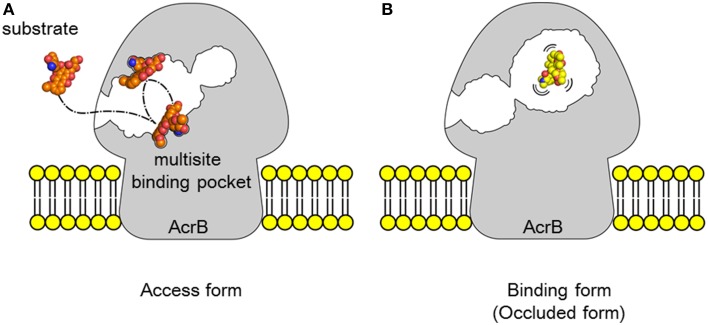
**Multisite-drug-oscillation hypothesis.(A)** *Access* stage. A drug is oscillating between multiple drug binding sites in the proximal pocket. **(B)** *Binding* stage. Most of LMMDs may be oscillating in the distal pocket as in the proximal pocket but HMMDs may be just occluded in the distal pocket without specific binding sites. Space-filling models in **(A,B)** show doxorubicin (orange) and erythromycin (yellow), respectively.

The multisite-drug-oscillation hypothesis may present a possible mechanism that explains the compatibility of high export efficiency with the apparently low affinity of substrate binding to specific sites (Figure 10). A recent molecular simulation study (Takatsuka et al., 2010) showed that the voluminous distal binding pocket of AcrB has binding sites for many drugs. These drugs have been classified as groove, cave and mixed binders. Interestingly, there are a number of possible binding sites for a single drug: however, a simulation study indicated that most of the possible binding sites were not equal to the actual site in the crystal. The results of this simulation study suggest that drug molecules oscillate in this voluminous binding pocket. In this mechanism, when the affinity of binding to each site is low, the total binding efficiency in the pocket may be high. The positions of the substrates that are “oscillating” in the binding pocket are difficult to detect in the crystal structure. Eicher et al. (2012) reported a doxorubicin dimer bound to the proximal pocket of AcrB. The low electron density of proximal doxorubicin molecules might indicate that doxorubicin molecules are not be dimers but instead a mixture of two different doxorubicin-binding AcrB trimers, i.e., each molecule binds at a different site in the proximal pocket. This result may indicate that the doxorubicin molecule oscillates between two binding sites in the proximal pocket and that the average electron density could be apparent in the crystal structure. Substrates tightly bound to one site can be detected, whereas substrates oscillating between multiple sites are rarely detectable in the crystal structure. The apparently fuzzy substrate recognition by AcrB may reflect such a multisite-drug-oscillation mechanism. HMMDs may be occluded without specific binding in the distal pocket (Figure 10B).

Rauch proposed the concept of “oscillating drug transporters” in order to explain multi-specificity of P-glycoprotein (Rauch, 2011). However, this “oscillating transporters” model is different from our “drug oscillation” hypothesis. “Oscillating transporters” means that transporter proteins oscillate between open/drug-accepting and closed/drug-expelling conformations in a membrane. Oscillating transporters stochastically catch substrates located in the membrane at the open form and then expels with the protein conformation change by oscillation. Specific drug binding sites are not assumed in oscillating transporter model. In contrast, our “multisite drug-oscillation” hypothesis does not mean transporter protein oscillation but means drug molecule oscillation between numerous drug-binding sites in the voluminous drug binding pockets. Both models can contribute multi-specificity, however, the selectivity of the substrates in the oscillating transporter model depends on the substrate solubility into the membrane. On the other hand, in the drug oscillation model, substrate selectivity depends on the affinity of each drug binding site and this model can explain the difference in the drug specificity between exporters, e. g., aminoglycoside antibiotics are not exported by AcrAB-TolC and MexAB-OprM systems but efficiently exported by MexXY system (Masuda et al., 2000; Elkins and Nikaido, 2002; Lau et al., 2014).

## Remote-conformational energy coupling

**Figure 11:**
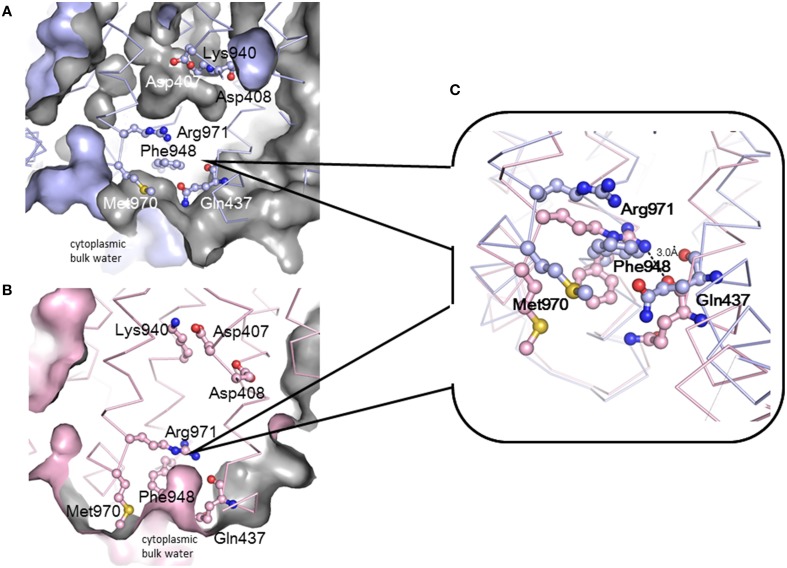
**Conformational changes in transmembrane proton relay residues during the functional-rotation cycle. (A,B)** show cut views of the lower transmembrane regions of the *binding* (blue) **(A)** and *extrusion* (pink) **(B)** monomers that have been drawn with Cα traces and the residues associated with proton relay depicted using ball and stick model. Blue and pink surfaces indicate the molecular surface of AcrB or the inside surface of the intramolecular void space. Gray color indicates the back of the surface or the surface of the intramolecular void space. **(C)** Magnified overlay of the vicinity of Arg971 in the *binding* and *extrusion* monomers. These figures were drawn based on the crystal structure of the asymmetric AcrB trimer using PyMol (Murakami et al., 2006).

Drug efflux by RND-type exporters is driven by the proton motive force (Thanassi et al., 1997; Li et al., 1998; Zgruskaya and Nikaido, 1999). Four essential charged residues, Asp407, Asp408, Lys940 (Lys939 in MexB), and Arg971, in addition to Thr978, are thought to form a transmembrane proton-relay network, based on findings from site-directed mutagenesis (Guan and Nakae, 2001; Su et al., 2006; Takatsuka and Nikaido, 2006). Asp407 and Asp408 in TM4 and Lys940 in TM10 form ion pairs in the transmembrane core (Figure 4C) (Murakami et al., 2002, 2006). TM4 and TM10 are located at the center of a 12 α-helix bundle. Arg971 is in the vicinity of the cytoplasmic surface of the membrane (Figure 11). Because this putative transmembrane proton-relay network is approximately 50 Å apart from the drug binding pocket, the energy coupling must reflect a remote-conformational coupling. In the asymmetric trimer, the ε-amino group of Lys940 is placed between the carboxyl groups of Asp407 and Asp408 and forms ion pairs in the *access* and *binding* monomers. In *extrusion* monomer, the side chain of Lys940 is twisted approximately 45° clockwise when viewed from the periplasm, and the ion pairs are abolished (Figure 4C insert) (Murakami et al., 2006), likely reflecting the protonation of the carboxyl group(s). Based on this side chain twisting, a TM bundle of the six N-terminal TMs (TM1-TM6) and a TM bundle of the six C-terminal TMs (TM7-TM12) are also twisted around each other. This bulky twisting movement in the transmembrane region occurs in conjunction with a series of conformational changes that result in the entrance closing, the exit opening and the drug being squeezed from the binding pocket at the *extrusion* stage. During the transition from the *extrusion* stage to the *access* stage, the deprotonation of carboxyl group(s) rebuilds the tripartite ion pairs. To drive drug export via the outside positive proton motive force, protonation during the transition from the *binding* to the *extrusion* stage should occur in the periplasm, and deprotonation during the transition from the *extrusion* to the *access* stage should occur in the cytoplasm. This result suggests an exchange mechanism involving the proton (or water) channel between the inward and outward configurations. Arg971 may be the “valve” that allows switching between inward and outward proton flow. As shown in Figure 11, the side chain of Arg971 is bent and is separated from the cytoplasmic bulk water by Phe948 and Met970 in the *binding* monomer (Figure 11A). However, this side chain faces the water-accessible void in the center of the transmembrane region that includes the Asp-Lys ion pairs. The void continues to a channel that is connected to the periplasm. Thus, Arg971 can receive a proton from Asp407 and/or Asp408 in the *binding* monomer. In contrast, in the *extrusion* monomer, the guanidino-pentanoic moiety of Agr971 is extended and slightly slanted downward, followed by the benzene ring of Phe948 and the methylthio-butanoic acid side chain of Met970 being pushed down and away from Arg971 (Figure 11C). As a result, the guanidino group of Arg971 is exposed to cytoplasmic bulk water (Figure 11B). At the *extrusion* stage, the void in the center of the transmembrane region is not present. Thus, Arg971 is an inside-facing structure from which protons can be released into the cytoplasm.

**Figure 12:**
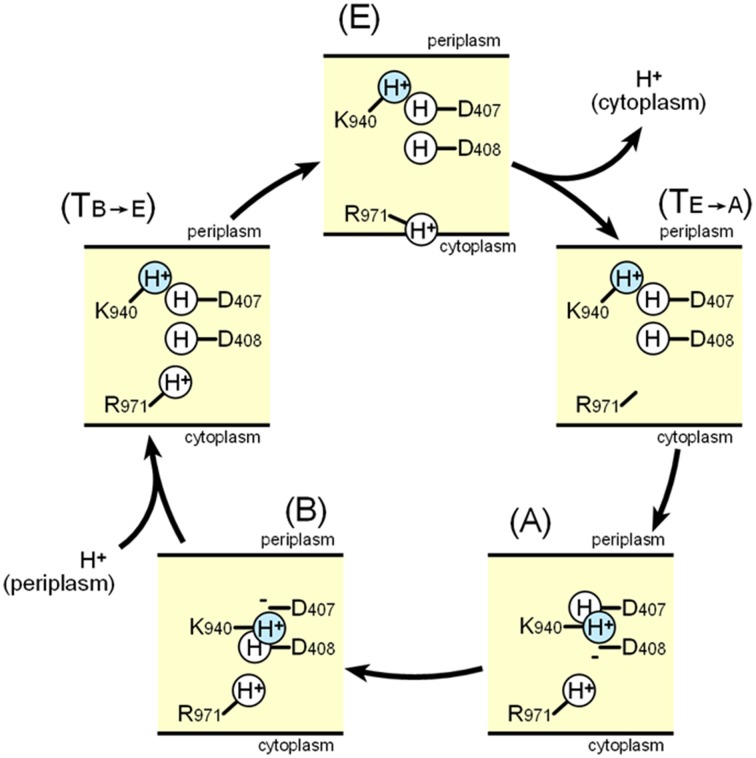
**Schematic diagram of the proton relay cycle in the transmembrane region of AcrB**. (E) *Extrusion* stage, (T_E-A_) putative transition state from the *extrusion* stage to the *access* stage. (A) *Access* stage, (B) *Binding* stage, (T_B-E_) putative transition stage from the *binding* stage to the *extrusion* stage. The blue color indicate permanently bound protons.

Figure 12 shows a potential scheme for proton translocation via the proton relay network based on observations of the crystal structure. Lys940 is postulated to be permanently protonated. All of the residues are protonated at the *extrusion* stage: thus the two aspartates are neutral, and the ion pair is abolished. At this stage, the side chain of Arg971 faces the cytoplasmic bulk water, and protons can be released into the cytoplasm via the proton motive force. Before the *access* stage, there is a transient state T_E−A._ The deprotonated guanidino side chain of Arg971 swings away from the bulk water toward the transmembrane core. Next, one proton bound to Asp408 is transferred to Arg971 probably via water molecules, because Arg408 is located closer to Arg971 than Asp407. As a result, Lys-Asp ion pairs are reformed at the *access* stage. Next, one proton bound to Asp407 is subsequently transferred to Asp408 during the *binding* stage. Subsequently, prior to the *extrusion* stage, an additional transient stage, T_B−E_ is needed, in which Asp407 is protonated from periplasm, tripartite ion pairs are abolished and the side chain of Lys940 swings away from the aspartate pair. There are some transferable proton residues, such as Asp566, Asp924, His338, and Glu346 (Eicher et al., 2014) on the periplasmic side of the transmembrane domain and the water accessible channel continues to the core ion pair region (Fischer and Kandt, 2011), potentially leading to the protonation of Asp407 from periplasm. Subsequently, the conformation returns to the *extrusion* stage via swinging of the protonated Arg971 side chain. In this scheme, one proton flows from the periplasm through Asp407, Asp408, and Arg971 to the cytoplasm in one cycle.

After determining the structures of the asymmetric AcrB trimers and identifying the transmembrane ion pair conformation changes during drug export (Murakami et al., 2006; Seeger et al., 2006; Sennhauser et al., 2007), molecular dynamics studies were performed to reveal the actuating mechanism of AcrB trimers, including an examination of the proton translocation pathway (Fischer and Kandt, 2011; Eicher et al., 2014). Water channels connecting the periplasm to the transmembrane core region were observed, including ion pairs involved at the *binding* and *access* stage, but these disappeared at the *extrusion* stage. The results of these analyses principally support the scheme shown in Figure 12. Eicher et al. (2014) reported changes in the orientation of Arg971 that may allow this amino acid to act as a valve for proton flow. Although Eicher et al. (2014) suggested that two protons are transported in one cycle (both Asp407 and Asp408 are deprotonated at the *binding* stage and protonated at the *extrusion* stage), it seems difficult to determine how Arg971 mediates the proton relay from the ion pairs to the cytoplasm, when carrying two protons in one cycle. Molecular dynamics simulations have their own limitations and do not necessarily reflect the actual phenomenon. It is likely that there is no need to change the simple one-way model in Figure 12 until experimental data conflicting with the scheme are reported or until detailed structures of the protons are determined.

## Tripartite structure of RND exporters and the drug sweeping/extrusion mode-switching hypothesis

The crystal structure of each component of the AcrAB-TolC complex has been determined (Koronakis et al., 2000; Murakami et al., 2002; Mikolosko et al., 2006): however, the complete tripartite crystal structure has not been solved. The crystal structure of a bi-partite complex of the inner membrane transporter and the adaptor protein of RND-type transporters has been solved but only for the CusBA complex (Su et al., 2011). Active forms of AcrB and TolC are most likely trimers (Koronakis et al., 2000; Murakami et al., 2002). The crystal structure of the bottom of native TolC channels is closed: however, this channel should remain open during drug export. The open form of the mutant TolC structure has been experimentally determined (Bavro et al., 2008; Pei et al., 2011). The crystal structures of AcrB and TolC suggest that both trimers directly dock to each other in a top-to-bottom manner (Murakami et al., 2002; Symmons et al., 2009) because the diameter and the shape of the funnel-like opening at the top of the AcrB trimer fit directly into the bottom of the open form of the TolC channel. This direct-docking model is experimentally supported by *in vivo* cross-linking between AcrB and TolC (Tamura et al., 2005; Weeks et al., 2010) and by the *in vitro* detection of the direct AcrB-TolC interaction without AcrA through surface plasmon resonance (Tikhonova et al., 2011).

**Figure 13:**
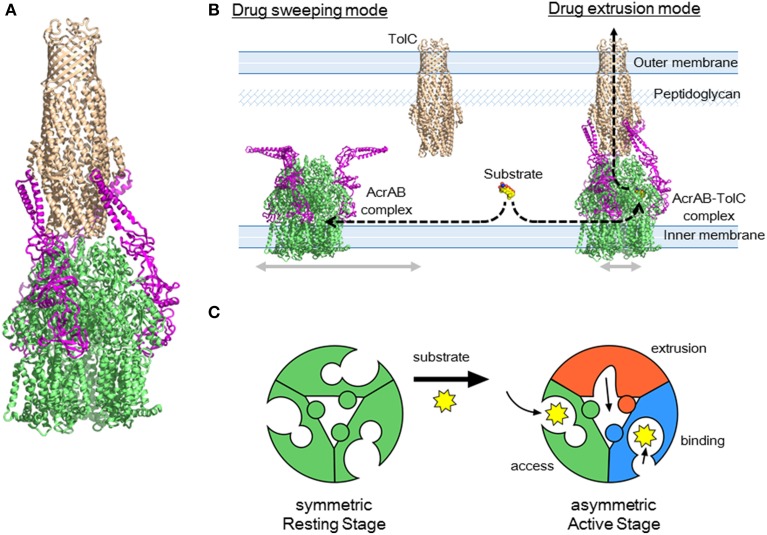
**AcrAB-TolC tripartite complex and the drug sweeping/extrusion mode-switching hypothesis. (A)** Currently postulated structure of the tripartite complex. The TolC (brown) trimer is directly docked with AcrB (green) trimer and three AcrA (pink) monomers are attached to the side. **(B)** Drug sweeping/extrusion mode-switching hypothesis. **(C)** AcrB trimers switching between the symmetric resting stage (left) and the asymmetric active stage (right).

Regarding AcrA, bi-partite AcrA-AcrB, and AcrA-TolC complexes are detected (Tikhonova and Zgurskaya, 2004; Touze et al., 2004), and AcrA is thought to recruit TolC to form a tripartite complex (Tikhonova et al., 2009). The AcrA structure has four domains: the α-hairpin, lipoyl, β-barrel, and MP (membrane proximal or β-roll) domains (Mikolosko et al., 2006; Symmons et al., 2009). Cross-linking between AcrA and AcrB showed a 1:1 stoichiometry (Symmons et al., 2009). AcrA-TolC cross linking (Lobedanz et al., 2007) and MexA-OprM (the *P. aeruginosa* homolog) cross linking (Ferrandez et al., 2012) also showed a 1:1 stoichiometry. Thus, the most likely model for the tripartite complex is that three AcrA molecules are attached to the TolC_3_-AcrB_3_ direct docking complex (Figure 13A) (Symmons et al., 2009). The α-hairpins of AcrA interact with TolC, and three other domains interact with the DN and PN2 domains of AcrB.

Although this AcrB_3_-AcrA_3_-TolC_3_ direct docking model seems probable on the basis of individual crystal structures and cross-linking experiments, this model was recently challenged in electron microscopic images of the AcrAB-TolC complex. Du et al. (2014) obtained *in vitro* images of the reconstituted AcrAB-TolC complex through cryo-electron microscopy and Kim et al. (2015) obtained images using transmission electron microscopy. The images showed the vertical length of the complex was 317 Å, which is significantly longer than that of the TolC-AcrB direct docking model (approximately 270 Å), indicating that the AcrA tube comprising the α-helical and lipoyl domains and a portion of the β-barrel domains is inserted between TolC and AcrB. The images also suggested a TolC_3_-AcrA_6_-AcrB_3_ stoichiometry. This indirect docking model is inconsistent with the CusBAC model, on the basis of the crystal structure of the CusBA bi-partite complex (Su et al., 2011). To obtain a tripartite complex, Du et al., used two kinds of linker proteins together: AcrA-AcrZ linker proteins and linker proteins in which AcrA is inserted within AcrB. The resultant complexes showed low activity *in vivo*. Kim et al., examined the AcrB-AcrA-AcrA linker protein. These linker proteins may force AcrA into the complex at a AcrA:AcrB stoichiometry being 2:1. Currently, there is no evidence indicating that such an indirect docking form is the active form *in vivo*. Using cryo-electron tomography, Trepout et al. (2010) reported an image of reconstituted MexA-OprM fitting to a 1:1 stoichiometry and suggested a two-step tripartite complex formation model. The indirect-docking complex may be an intermediate step in the formation of an active complex. Regarding the stoichiometry of AcrA in the complex, we recently observed that the AcrB-AcrA one-to-one linker protein exhibits complete activity in the AcrA/AcrB-deficient base (unpublished observation), suggesting that a 1:1 stoichiometry of AcrA to AcrB is sufficient for drug export. Controversy regarding the stoichiometry and the construction of the tripartite complex will continue until a high-resolution crystal structure is determined.

RND-type multidrug exporters take up substrates from the outer layer of the inner membrane and/or periplasm from dual drug entry points (Sennhauser et al., 2007; Husain et al., 2011; Nakashima et al., 2011). Considering the high efficiency of drug export and the relatively low-level intrinsic expression of RND transporters, each transporter should rapidly sweep the inner membrane through lateral diffusion. However, the lateral movement of the trans-periplasm complex such as AcrAB-TolC is prevented by a peptidoglycan mesh. To both rapidly sweep for substrates and efficiently export these into the TolC channel, it may be necessary to switch between a horizontally-diffusing drug-sweeping mode and the TolC-fixed drug-extrusion mode. Because TolC is a multifunctional outer membrane protein that interacts with a number of inner membrane transporters, complex formation would need to be tentative for optimal functioning (Zgruskaya, 2009).

In the sweeping mode, the inner membrane transporter alone or with adaptor proteins may move laterally via Brownian motion in the lipid bilayer region (Figure 13B). In the absence of substrates, inner membrane transporters are likely symmetric comprising the three monomers being the same *access*-like structure (Figure 13C). When adaptor proteins are attached to the moving transporters, the α-helical moiety is likely to be bent downward. Although the bent conformation of AcrA has not been previously described, this bent-downward structure may be easily accommodated by AcrA because the AcrA structure shows high flexibility between subdomains (Vaccaro et al., 2006). When a substrate binds one of the monomers, the conformation of the trimer changes to the asymmetric form. As a result, AcrA is primed for the recruitment of TolC, followed by tentative tripartite complex formation. Immediately after drug export, the tripartite complexes dissociate. When the substrate concentration is high, the complex may continuously take up substrates without dissociating. This sweeping and extrusion mode exchange hypothesis seems to provide reasonable explanation for the high efficiency of drug efflux through the trans-periplasm complex. FDAP (fluorescence decay after photoconversion) analysis using PA-GFP (photoactivatable-GFP)-labeled AcrB showed the lateral movement of AcrB is more rapid when AcrB is expressed in the *acr*B/*tol*C-deficient cells than in the *acr*B-deficient cells. AcrB movement is slowed in the presence of proximal binding drugs (unpublished observation). Thus, AcrB is an exciting future target for investigations into how and when tripartite complexes form and what their physiological role(s) is.

## Specific recognition of inhibitors

**Figure 14:**
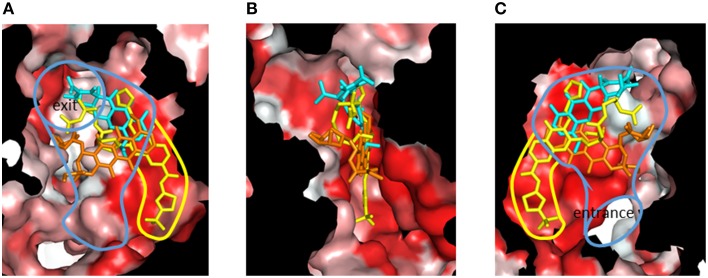
**Cut view of the distal pocket of the AcrB *binding* monomer in complex with the inhibitor ABI-PP (yellow)**. The bound minocycline (cyan) and doxorubicin (orange) are overlaid. **(A)** View toward the exit. **(B)** View looking down the hydrophobic trap (90° rotated from **(A)** around the vertical axis). **(C)** View toward the entrance (90° rotated from **(B)** around the vertical axis). The red color indicates Eisenberg's hydrophobicity scale. The blue and yellow curves indicate the drug translocation channel and the hydrophobic trap, respectively.

**Figure 15:**
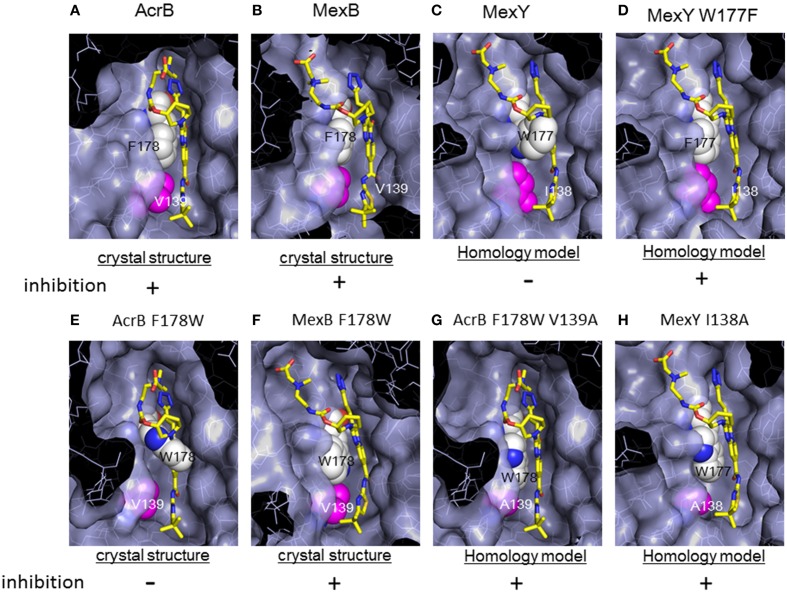
**Magnified view of the ABI-PP binding site depicted as a surface model. ABI-PP is depicted in a stick model**. F178 and W177 are depicted using a white space-filling model. V139, I138, and mutated Ala are depicted shown in magenta in the space-filling model. The symbols + and − indicate inhibition or the lack of inhibition by ABI-PP, respectively. **(A,B,E,F)** are crystal structures, and **(C,D,G,H)** are homology models. **(A)** ABI-PP-binding AcrB, **(B)** ABI-PP-binding MexB, **(C)** MexY overlapping with ABI-PP. **(D)** MexY W177F overlapping with ABI-PP, **(E)** AcrB F178W overlapping with ABI-PP, **(F)** ABI-PP-binding MexAB F178W, **(G)** AcrB F178W V139A overlapping with ABI-PP, **(H)** MexY I138A overlapping with ABI-PP.

Although RND-type transporters display a broad substrate recognition spectrum, these proteins show strict specificity for some inhibitors. Pyridopyrimidine derivatives are good inhibitors of AcrB and MexB without toxic effects: however, these compounds do not inhibit MexY (Yoshida et al., 2007). The narrow spectrum of pyridopyrimidines limits the clinical usefulness of these molecules. The structural basis of inhibitor specificity has been revealed through an analysis of the inhibitor-bound crystal structure of AcrB and MexB (Nakashima et al., 2013). The pyridopyrimidine derivative ABI-PP binds to the distal pocket of AcrB and MexB. The hydrophobic tail of ABI-PP is inserted into a narrow hydrophobic pit branching off the substrate translocation path (Figure 14). The binding site of the relatively hydrophilic moiety of ABI-PP overlaps with the minocycline and doxorubicin binding sites. The branched pit is inconsistent with a hydrophobic trap in the distal binding pocket (Vargiu et al., 2011). The F610A mutation in this pit caused slip-in of substrates into this pit, resulting in decreased export activity. Phe178 is located at the edge of this pit in AcrB and MexB, and the benzene ring of this amino acid forms π - π interactions with the pyridopyrimidine bicyclic ring, thereby stabilizing ABI-PP binding (Figures 15A,B). The inhibitory activity of ABI-PP is based on strong binding to this pit, which terminates the functional-rotation cycle because this pit has to become closed off for transition to the *extrusion* stage.

However, in a homology model of MexY, the corresponding position is occupied by tryptophan (Trp177), from which the bulky indolyl side chain protrudes into the pit and sterically hinders ABI-PP binding (Figure 15C). When Trp177 of MexY was replaced with phenylalanine by site-directed mutagenesis (Figure 15D), the resultant W177F mutant of MexY showed a susceptibility to ABI-PP similar to that observed for AcrB without the loss of drug export activity. In contrast, when Phe178 of AcrB was replaced with tryptophan, the resultant AcrB F178W mutant showed resistance to ABI-PP similar to MexY. The crystal structure of the AcrB F178W mutant was solved and the indolyl side chain of Trp178 protruded into the pit (Figure 15E). Thus, ABI-PP specificity is determined by the bulkiness of the side chain at position 178 or 177: however, the MexB F178W mutant remains sensitive to ABI-PP. The crystal structure of the ABI-PP-bound MexB F178W mutant showed that the bulky indolyl side chain is accommodated in parallel to the wall of the pit without projection, thereby contributing to stable binding through π - π interactions with the pyridopyrimidine ring (Figure 15F).

An *in silico* simulation revealed that the parallel-to-wall arrangement of the indolyl moiety of Trp178 in AcrB is impossible due to steric hindrance from Val139. The pit in MexB is slightly larger than that in AcrB: thus, the parallel arrangement of the indolyl moiety of Trp178 is permitted in MexB but not permitted in AcrB. Ile138 of MexY also sterically hinders the parallel arrangement of the side chain of Trp177. To confirm this prediction, the AcrB F178W V139A double mutant and the MexY I138A mutant were constructed (Figures 15G,H). These mutants showed an ABI-PP-sensitive phenotype similar to wild-type AcrB. Thus, the specificity for pyridopyrimidine derivatives is determined by the fit to the hydrophobic pit in the distal binding pocket. The ABI-PP binding structures of AcrB and MexB are the first examples of inhibitor-binding structures of multidrug efflux transporters in physiologically active asymmetric forms. These observations provide information for the development of universal inhibitors that inhibit AcrB, MexB and MexY, through virtual screening and structure-based drug design.

## Concluding remarks and future perspectives

The molecular mechanisms of multidrug recognition and export by RND-type drug exporters have been revealed via crystal structure determinations over past decade. Multidrug recognition is based on multisite drug-binding in voluminous binding pockets. The presence of two voluminous drug-binding pockets, proximal and distal, significantly expands the substrate specificity of these exporters. Multiple-entrances allow the export of both hydrophobic and hydrophilic compounds. Drug efflux is mediated through functional-rotation mechanism in which three monomers undergo a strictly coordinated sequential conformational change cycle of *access, binding* and *extrusion*. During the functional-rotation cycle, no two monomers display the same conformation. The substrates are transported from the entrance to a proximal pocket and then to a distal pocket and finally to a funnel-like exit through the peristaltic motion of the AcrB porter domain. Drug export is driven by the proton motive force via a remote-conformational coupling mechanism. The proton relay cycle in the transmembrane region strictly couples with the functional-rotation cycle in the porter region. Specific inhibitors bind tightly to the deep hydrophobic pit of the multisite drug binding pocket.

Future studies should address questions concerning why it is difficult to identify most bound substrates in the asymmetric structure and how the immobile trans-periplasmic exporter efficiently ejects substrates before they enter the cytoplasm. The former question could be answered by the multisite-drug-oscillation hypothesis, which is consistent with a broad binding specificity and highly efficient export. The latter question could be addressed by the sweep (moving) and export (fixed) mode-switching hypothesis, which is consistent with the need for tentative formation of a tripartite complex. However, these hypotheses lack experimental evidence. Addressing these questions is an exciting challenge, which will involve protein dynamics studies and crystal structure determinations of the tripartite complex.

### Conflict of interest statement

The authors declare that the research was conducted in the absence of any commercial or financial relationships that could be construed as a potential conflict of interest.
